# The cGAS/STING Pathway—A New Potential Biotherapeutic Target for Gastric Cancer?

**DOI:** 10.3390/jpm14070736

**Published:** 2024-07-09

**Authors:** Mengxiang Tian, Shuai Zhang, Fengbo Tan

**Affiliations:** 1 Department of General Surgery, Xiangya Hospital, Central South University, 87 Xiangya Road, Changsha 410017, China; 12218560@zju.edu.cn (M.T.); fengbotan@csu.edu.cn (F.T.); 2 National Clinical Research Center for Geriatric Disorders, Xiangya Hospital, Central South University, Changsha 410017, China

**Keywords:** cGAS-STING, gastric cancer, innate immune response, cancer biotherapy, tumor immunosuppressive microenvironment

## Abstract

Gastric cancer ranks among the top five deadliest tumors worldwide, both in terms of prevalence and mortality rates. Despite mainstream treatments, the efficacy in treating gastric cancer remains suboptimal, underscoring the urgency for novel therapeutic approaches. The elucidation of tumor immunosuppressive microenvironments has shifted focus towards cancer biotherapeutics, which leverage the patient’s immune system or biologics to target tumor cells. Biotherapy has emerged as a promising alternative for tumors resistant to traditional chemotherapy, radiation, and immunotherapy. Central to this paradigm is the cGAS-STING pathway, a pivotal component of the innate immune system. This pathway recognizes aberrant DNA, such as that from viral infections or tumor cells, and triggers an immune response, thereby reshaping the immunosuppressive tumor microenvironment into an immune-stimulating milieu. In the context of gastric cancer, harnessing the cGAS-STING pathway holds significant potential for biotherapeutic interventions. This review provides a comprehensive overview of the latest research on cGAS-STING in gastric cancer, including insights from clinical trials involving STING agonists. Furthermore, it assesses the prospects of targeting the cGAS-STING pathway as a novel biotherapeutic strategy for gastric cancer.

## 1. Backgrounds

Gastric cancer stands as one of the most pervasive malignancies globally, characterized by its significant morbidity and mortality rates, posing a formidable challenge to public health worldwide. Ranked as the fifth most common cancer globally, gastric cancer ranks third in cancer-related deaths due to its aggressive nature [1,2]. Its primary risk factors encompass Helicobacter pylori infection, dietary patterns, smoking, Epstein-Barr virus (EBV) infection, and genetic predispositions [3]. The burden of gastric cancer is particularly pronounced in East Asia and South America. In China, digestive system cancers contribute to 41.6% of new cancer cases and 49.3% of cancer-related deaths, with gastric cancer representing a substantial portion of these statistics [4,5,6]. Recent guidelines from the National Comprehensive Cancer Network (NCCN) emphasize the importance of precision therapy alongside conventional treatments such as surgical resection, radiotherapy, chemotherapy, targeted therapy, and immunotherapy. Precision therapy is particularly pertinent for cases of gastric cancer with poor prognoses [7,8]. Therefore, the quest for novel targets in gastric cancer treatment assumes paramount importance, aiming to address the prevalent issue of resistance to immune checkpoint inhibitor monotherapy among gastric cancer patients.

Cancer biotherapy harnesses biological agents to trigger the host’s innate anti-tumor response, effectively impeding tumor cell proliferation. The human immune system assumes a pivotal role in the initiation, progression, and metastasis of malignant tumors, exerting its tumor-suppressing effects by activating robust anti-tumor responses. This immune-mediated mechanism is closely linked to the prognosis of various cancer therapies [9]. Currently, biotherapeutic interventions targeting T cells have demonstrated promising therapeutic outcomes against both solid tumors and hematological malignancies [10]. Consequently, the prospect of achieving durable tumor treatment and control via modulation of the innate immune system holds significant promise [11,12].

The cyclic GMP–AMP synthase (cGAS)–stimulator of interferon genes (STING) pathway serves as a pivotal mediator of innate immune responses, uniquely activated by the detection of DNA from both foreign microbial sources and the host itself. This pathway plays a critical role in diverse physiological processes, including inflammation, stress responses, autoimmune disorders, and tumorigenesis [13,14,15,16].

This review outlines the expression of cGAS-STING in gastric cancer and its association with key genetic markers related to gastric cancer, elucidating its potential therapeutic implications in this context. Furthermore, it delves into future research prospects pertaining to the transduction of the cGAS-STING pathway and its pivotal role in facilitating the clinical translation of novel therapies for gastric cancer.

## 2. Activation Mechanism of cGAS-STING Pathway

Exogenous viruses, bacteria, sterile inflammation, tumors, chromosome instability, and chemotherapy can all inflict cellular damage, leading to the release of double-stranded DNA (dsDNA) [17]. Ordinarily confined within the nucleus and mitochondria, the presence of dsDNA in the cytoplasm serves as a damage-associated molecular pattern (DAMP). Consequently, dsDNA that escapes lysosomal degradation becomes implicated in the initiation and progression of various inflammatory conditions and tumors through the activation of the cGAS-STING signaling pathway.

cGAS, short for “cyclic GMP-AMP synthetase”, is situated within the cytoplasm, where it detects the presence of dsDNA—an anomaly in this cellular compartment—and catalyzes the conversion of GTP and ATP into “cyclic GMP-AMP” (cGAMP) [18,19]. This enzymatic process involves two functional domains within the cGAS protein: the N-terminal nucleotide transferase domain (NTase domain) and the C-terminal nucleotide transferase domain (CTD). The NTase domain primarily recognizes and binds dsDNA molecules, whereas the CTD facilitates the synthesis of cGAMP from ATP and GTP substrates. Notably, the resulting cGAMP, containing two phosphodiester bonds, exhibits heightened affinity for STING [20,21]. Upon cGAMP binding to STING, an intracellular immune response is triggered, activating pathways that culminate in the production of interferon and other immune-related molecules essential for combating infections [22]. Hence, cGAS functions as an “accelerator” for the immune system, recognizing both endogenous and exogenous DNA to initiate immune responses. This mechanism fortifies the body’s “attack force” against tumors and pathogenic microorganisms.

STING (Stimulator of Interferon Genes) is a transmembrane protein consisting of 379 amino acid residues, predominantly localized within the endoplasmic reticulum (ER). Its structure comprises distinct functional domains crucial for its role in cellular signaling. The N-terminal region encompasses the first 137 amino acid residues, forming a transmembrane domain that anchors STING to the ER membrane. Residues 138 to 340 constitute the cyclic dinucleotide binding domain, essential for STING’s interaction with cGAMP and initiating downstream signaling cascades. The remaining residues (341 to 379) constitute the C-terminal tails (CTT), potentially modulating STING function or interactions [23,24,25]. The ligand-binding domain of the STING protein is located in its C-terminal domain and is responsible for recognizing and binding intracellular cyclic dinucleotides (such as cGAMP). Upon binding with cGAMP, the ligand-binding domain of STING undergoes a conformational change, leading to the dissociation of the CTT. Subsequently, activated STING undergoes aggregation and translocates to the Golgi apparatus via the endoplasmic reticulum Golgi intermediate compartment (ERGIC) [26]. Following activation, STING recruits and activates tank-binding kinase 1 (TBK1) via a specific binding motif (PLPLRT/SD) within its CTT. TBK1, an inhibitor of kappa B kinase (IKK)-related kinase, upon binding to STING, phosphorylates STING itself, augmenting its activity and facilitating prolonged signal transduction. Concurrently, TBK1 phosphorylates interferon regulatory factor 3 (IRF3), a pivotal transcription factor in the interferon signaling pathway [27,28]. Phosphorylated IRF3 undergoes conformational changes, enabling its dimerization and nuclear translocation. This process enhances the transcription of interferon (IFN) and other immune-related genes, thereby bolstering the cellular immune response [29].

In addition to the classical IFN pathway, the nuclear factor kappa B (NF-kB) serves as another crucial downstream effector of STING, contributing to the regulation of inflammatory and immune responses [30,31]. Recent investigations have unveiled that STING activation triggers the activation of TGF-beta-activated kinase 1 (TAK1) and I kappa B kinase (IKK) complexes, culminating in the phosphorylation and subsequent degradation of I kappa B [32,33]. Consequently, the degradation of IkB permits NF-kB to translocate into the nucleus, initiating NF-kB-mediated gene transcription. This transcriptional activation induces the production of inflammatory factors vital for sustaining immune and inflammatory responses.

**Figure 1:**
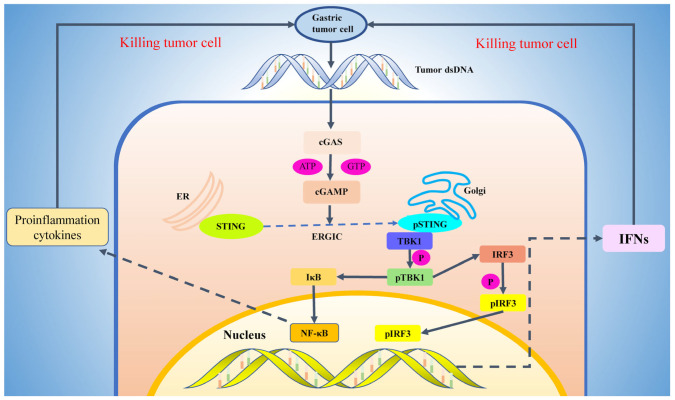
cGAS-STING pathway in tumors. In tumors, where cells tend to accumulate significant DNA damage, cGAS plays a pivotal role by catalyzing the production of cGAMP upon the detection of tumor-derived double-stranded DNA (dsDNA). Subsequently, cGAMP binds to the STING protein located on the endoplasmic reticulum (ER), initiating a cascade of events. Activated STING is translocated from the ER to the Golgi apparatus via the endoplasmic reticulum-Golgi intermediate compartment. Once activated, STING triggers the activation of key signaling molecules such as TBK1 (TANK-binding kinase 1) and IRF3 (Interferon Regulatory Factor 3). This activation culminates in the production of transcription factors NF-κB and interferon upon their translocation into the nucleus. Ultimately, these molecular responses orchestrate the immune-mediated destruction of tumor cells, thereby contributing to tumor suppression. cGAS cyclic GMP–AMP synthase; STING stimulator of interferon genes; dsDNA double-stranded DNA; cGAMP cyclic GMP-AMP; ATP Adenosine Triphosphate; ER Endoplasmic reticulum; ERGIC endoplasmic reticulum Golgi intermediate chamber; TBK1 tank-binding kinase 1; IKK inhibitor of kappa B kinas; IRF3 interferon regulatory factor 3; IFN interferon; NF-kB nuclear factor kappa B. The role of the cGAS-STING signaling pathway in tumor biotherapy.

Upon completion of the immune response, the STING protein undergoes ubiquitination. The ubiquitinated STING is then recognized and transported to the lysosome, where it undergoes degradation, effectively curtailing excessive immune responses [34] (Figure 1).

## 3. The Role of cGAS-STING Signaling Pathway in Tumor Biotherapy

The tumor microenvironment (TME) is a complex and heterogeneous milieu comprising various cell types aside from tumor cells, encompassing fibroblasts, stromal cells, immune cells, and the vascular system. Within this intricate landscape, the TME fosters tumor growth and progression through mechanisms such as angiogenesis induction, the establishment of an immunosuppressive milieu, and the facilitation of tumor cell evasion. These processes significantly curtail the efficacy of conventional tumor treatments like surgery, radiotherapy, and chemotherapy [35,36]. Notably, the activation of the cGAS-STING signaling pathway assumes a pivotal role in tumor biotherapy.

Upon activation of the cGAS-STING signaling pathway, a significant production of interferons (IFNs) ensues. IFNs represent a pivotal class of immunomodulatory molecules crucial for anti-tumor immunity. They facilitate several key processes: Firstly, IFNs promote the recognition and processing of tumor-associated antigens (TAAs) by antigen-presenting cells, such as dendritic cells, initiating the activation of tumor-specific T cells for the targeted elimination of tumor cells. Additionally, IFNs bolster the recruitment and activation of immune effectors like T cells and natural killer cells within tumor tissues, augmenting their tumor-killing capabilities and fortifying immune responses. Furthermore, IFNs orchestrate a shift in the tumor microenvironment by diminishing the production of immunosuppressive factors, such as anti-inflammatory cytokines and immunosuppressive cytokines, while amplifying the generation of immunostimulatory factors like pro-inflammatory cytokines and chemokines. This recalibration enhances immune cell functionality against tumors [37,38]. Clinical investigations have substantiated the correlation between IFN-γ-induced heightened levels of infiltrating T cells and improved overall survival in patients afflicted with colorectal cancer [39,40]. Nonetheless, tumors deploy various strategies to thwart IFN-mediated immune responses. They may inhibit IFN formation via the JAK-STAT signaling pathway or release inhibitory factors. Moreover, tumors secrete chemokines like lectins to curtail the recruitment of T cells to the tumor microenvironment, thereby evading immune surveillance and minimizing the likelihood of immune system attacks [41,42].

**Figure 2:**
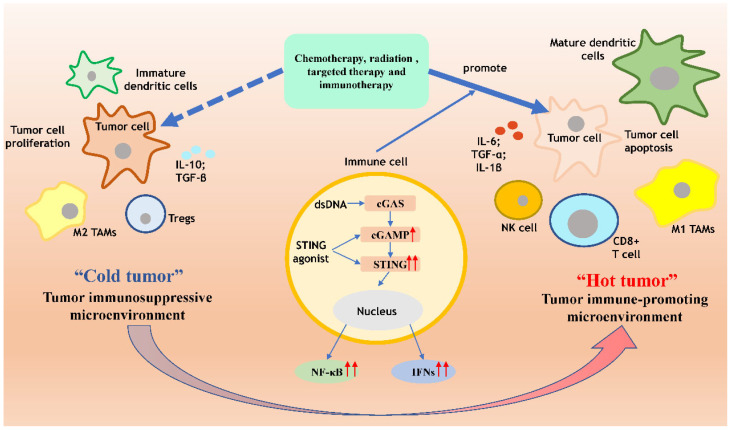
cGAS-STING can transform a cold tumor into a hot tumor and promote tumor cell apoptosis. The immunosuppressive microenvironment comprises primarily immature dendritic cells, immunosuppressive Treg cells, and M2 tumor-associated macrophages, alongside chemical factors such as IL-10 and TGF-β released by tumor cells. These immunosuppressive elements collaborate to curtail the efficacy of targeted therapies like traditional chemotherapy and radiotherapy, thereby fostering tumor progression, dissemination, and evasion of immune surveillance. Through the release of IFNs and NF-κB, the cGAS-STING pathway facilitates the maturation of dendritic cells, the recruitment of cytotoxic CD8+ T cells, the polarization of M2 tumor-associated macrophages toward the M1 phenotype, and the augmentation of inflammatory cytokine secretion. Consequently, the immunosuppressive microenvironment undergoes a transition into an immune-supportive milieu, thereby enhancing the therapeutic outcomes against tumors and prompting tumor cell apoptosis. cGAS cyclic GMP–AMP synthase; STING stimulator of interferon genes; IFN interferon; NF-kB nuclear factor kappa B; NK cells natural killer cells; TAM tumor-associated macrophages. The cGAS-STING pathway in gastric cancer.

The cGAS-STING signaling pathway has the capability to reverse this detrimental phenomenon. For instance, research has unveiled that activating STING signaling via constitutively active STING in both human and murine colorectal cancer (CRC) models can induce endogenous interferon signaling within CRC cells, even in those with intact mismatch repair (MMR) signaling. This activation leads to the initiation of local interferon signaling in cancer cells, the recruitment of antigen-presenting cells, the mobilization of effector lymphocytes, and enhances the responsiveness of previously “cold” tumor microenvironments (TMEs) to in vivo immune checkpoint inhibitor (ICI) therapy [43]. Thus, the cGAS-STING signaling pathway has the potential to counteract the inhibitory effects exerted by tumor cells on interferons, by continuously releasing substantial amounts of interferons. This transformation effectively converts a “cold tumor” into a “hot tumor”, thereby shifting the tumor microenvironment from an immunosuppressive state to an immune-promoting one. Consequently, this process not only impedes tumor development and metastasis but also enhances the efficacy of other therapeutic modalities [44,45,46] (Figure 2).

## 4. The cGAS-STING Pathway in Gastric Cancer

As mentioned earlier, the primary risk factors for gastric cancer include infections by Helicobacter pylori and Epstein-Barr virus (EBV). As exogenous pathogens, their DNA can bind to cGAS and activate the cGAS-STING signaling pathway [47]. Researchers observed that after 20 weeks of Helicobacter pylori infection, immunohistochemical analysis revealed a significant increase in STING protein expression in the gastric epithelial cells of mice [48]. Additionally, quantitative PCR results showed elevated mRNA expression levels of interferons (IFNs) and the downstream inflammatory factor IL-6 in gastric tissue, confirming the activation of the cGAS-STING pathway by Helicobacter pylori infection [48]. Building upon this observation, further investigations demonstrated significantly higher STING protein expression in normal tissues adjacent to cancer compared to tumor tissues in surgical resection samples from clinical gastric cancer patients. Patients exhibiting lower STING protein expression levels in gastric tumor tissues exhibited more advanced TNM stages and lower overall survival rates [48]. Regarding EBV infection, STING expression was notably higher in the surgical specimens of patients with EBV-positive gastric cancer compared to EBV-negative cases. Interestingly, patients with STING-positive expression demonstrated better overall survival rates regardless of EBV infection status [49]. Moreover, a predictive model based on cGAS-STING pathway-associated genes (CSRs) was developed to forecast overall survival in gastric cancer patients using bioinformatics techniques applied to The Cancer Genome Atlas (TCGA) and Gene Expression Omnibus (GEO) databases [50]. Additionally, a study by Gao et al. identified prognosis and immunotherapy biomarkers (M2GO), including STING downstream molecules TBK1 and IRF3, utilizing transcriptome sequencing data from clinical gastric cancer patients with ovarian metastasis, which were externally validated using the TCGA-STAD database. Patients with lower M2GO expression levels exhibited relatively poorer prognoses [51]. Furthermore, Japanese researchers found that STING protein expression levels and CD8+ T cell infiltration in gastric cancer samples from HER2-positive patients were significantly lower compared to HER2-negative patients. Inhibition of HER2 resulted in upregulated expression levels of STING protein and CD8+ T cell infiltration, suggesting that HER2 positivity may promote a tumor immunosuppressive microenvironment in gastric cancer by inhibiting STING expression and CD8+ T cell infiltration, thereby worsening patient prognosis [52] (Table 1).

While there are currently no ongoing clinical trials investigating STING agonists specifically for gastric cancer, promising results from experimental studies conducted in cell and animal models instill confidence in their potential efficacy. In gastric cancer cell lines, for instance, metformin has demonstrated the ability to not only inhibit the mTOR1 signaling pathway via classical AMPK-dependent or independent mechanisms but also to exhibit anti-tumor effects by activating the cGAS-STING pathway [55]. Similarly, in both gastric cancer cells and mouse-transplanted tumor models, Anlotinib has shown efficacy in reducing tumor proliferation and metastasis by activating the cGAS-STING signaling pathway [53]. Additionally, it has been observed to enhance the anti-tumor efficacy of anti-PD-L1 therapy. It is noteworthy that while gastric cancer cells undergoing chemotherapy, radiotherapy, or other stress responses often experience cell damage or apoptosis, leading to the release of endogenous dsDNA into the cytoplasm and activating anti-tumor immune responses via the cGAS-STING pathway, these cells may also evade this immune surveillance through various mechanisms, such as reducing their DNA sensing function. However, combination therapy may counteract this effect. In studies utilizing Mouse Forestomach Carcinoma (MFC) cells and MFC tumor-bearing mice, it was found that radiotherapy significantly upregulated the gene expression of the STING signaling pathway, as well as the expression of PD-1/PD-L1 [54]. Additionally, Hosseinzadeh et al.’s recent study found that STING agonists did not directly affect the viability of gastric cancer cells. However, the combined treatment of anti-PD-1, IFN-γ, and STING agonists increased the apoptosis rate in the treatment group. This further suggests that the combination of IFN-γ, anti-PD-1, and STING agonists could provide a promising approach for gastric cancer immunotherapy [56].

## 5. Types of STING Agonists and Their Related Clinical Trials

STING agonists, heralded as prominent agents in contemporary tumor biotherapy, function by stimulating the release of interferons (IFNs) through the activation of STING. This activation fosters an immune-reactive tumor microenvironment, reverses immune tolerance, amplifies the efficacy of immunotherapy, and constrains tumor progression. Broadly categorized, STING agonists comprise cyclic dinucleotides (CDN) and non-cyclic dinucleotides (NCDN) [57,58] (Table 2).

CDN agonists represent the natural ligands of STING and were among the earliest STING agonists discovered. These cyclic dinucleotides, such as 3′3′-cGAMP and 2′3′-cGAMP, are synthesized by bacteria or fungi and are released into host cells during infection to activate the STING pathway [62]. However, initial CDN candidates exhibited poor in vivo experimental results, prompting the development of novel CDN drugs [59,60]. Currently, a diverse array of CDN STING agonists has advanced into clinical trials. For instance, MIW815, also known as ADU-S100 and developed by Aduro Biotech, belongs to the cyclic GMP-AMP (cGAMP) class of STING agonists [63]. Notably, MIW815 has demonstrated efficacy in inhibiting colon cancer peritoneal metastasis in animal models [64]. Clinical trials have further shown that MIW815, either alone or in combination with the PD-1 inhibitor Spartalizumab, improves outcomes in patients with advanced metastatic lymphoma [64,65]. Similarly, Merck has developed MK-1454, a cGAMP STING agonist, which, when combined with Pembrolizumab, enhances outcomes in patients with advanced solid tumors or lymphomas [66]. Furthermore, BMS has developed BMS-986301, based on IFM Therapeutics’ research [67]. Although clinical trial results are pending, the significant efficacy observed in CRC animal models when combined with PD-1 inhibitors has instilled confidence in researchers. Additionally, Spring Bank Pharmaceuticals has developed SB-11285, currently undergoing clinical trials in combination with atezolizumab, an anti-PD-L1 monoclonal antibody, in patients with advanced solid tumors [68].

When compared to CDN agonists, NCDN agonists offer potential advantages, including oral delivery feasibility and lower-cost production [69]. Despite the promise of the most classic NCDN agonist, DMXAA (Vadimezan), in numerous clinical trials, its failure stemmed from an inability to effectively engage with human STING [70]. Consequently, there has been a shift towards developing NCDN agonists specifically targeting human STING, exemplified by compounds like GSK-3745417, TAK-676, SNX281, and MK-2118. These novel agents are currently undergoing evaluation in combination with other drugs, such as Pembrolizumab, in Phase I trials for advanced solid tumors or non-small cell lung cancer [58,71] (Table 3).

## 6. Application of Nano Drug Delivery Technology

While STING agonists exhibit significant promise in eliciting anti-tumor immunity, their clinical application is hindered by several limitations, including a short serum half-life, inadequate cytoplasmic delivery, and tissue specificity [72]. Nano drug carrier technology, leveraging nanomaterials to encapsulate drugs into nanoparticles, offers a solution to enhance drug delivery, release, and targeting, thereby addressing these challenges in the clinical application of STING agonists [73,74]. Various nanocarrier drug delivery technologies have been developed to facilitate the delivery of STING agonists. These include hydrogels, self-assembly systems, exosomes, liposome preparations, and polymer nanoparticles, all of which have found extensive application in STING agonist delivery systems [75]. For instance, liposome preparation technology has been utilized to encapsulate hydrophilic and negatively charged STING agonists within the hydrophobic core of micro-nanoparticles. This approach not only prolongs the half-life of STING agonists but also ensures more effective cytosolic delivery [76,77]. Additionally, exosome and polymer nanoparticle delivery techniques have been employed to enhance the low cell membrane penetration efficiency of STING agonists, thereby improving therapeutic efficacy [78,79,80,81].

## 7. Conclusions and Discussions

As an immune surveillance mechanism, the cGAS-STING pathway primarily elicits the production of interferons and other immune regulatory factors by detecting abnormalities in endogenous and exogenous DNA, including viral infections and DNA damage in tumor cells. Consequently, it promotes an anti-tumor immune response and assumes a crucial role in tumor biotherapy.

Several clinical studies have found that STING expression is downregulated in gastric cancer patients, and the loss of STING is positively correlated with tumor progression (possibly due to a reduced DNA sensing function), thereby affecting anti-tumor immune responses and cancer progression [48]. These studies also highlight the prognostic and therapeutic significance of the cGAS-STING signaling pathway in gastric cancer. Specifically, the expression level of STING correlates positively with the prognosis of gastric cancer patients, particularly those with Helicobacter pylori or EBV infection, as well as HER2-positive cases. Although clinical trials investigating STING agonists for gastric cancer are currently lacking, encouraging therapeutic effects have been observed with agents like metformin and Anlotinib in animal models through STING activation. Given the rapid advancements in the nanomaterial delivery of STING agonists and promising results from clinical trials evaluating STING agonists in combination with other anti-tumor agents for advanced solid tumors or lymphomas, we advocate for the utilization of STING agonists in conjunction with other therapies—such as chemotherapy, radiotherapy, anti-angiogenic therapy, and immunotherapy—to enhance the clinical efficacy of anti-tumor therapy for gastric cancer.

However, it must be acknowledged that although studies involving clinical samples, experimental animal models, and in vitro cell assays have demonstrated that low STING expression is associated with poor prognosis in gastric cancer, STING activation could be a promising therapeutic strategy to inhibit tumor growth by enhancing immune responses. Nonetheless, STING agonists might trigger systemic inflammatory responses, leading to increased side effects. Additionally, there could be significant variability in patient responses to STING agonists, necessitating further research to identify which patients are most likely to benefit from this therapy. Overall, targeting STING in the treatment of gastric cancer is a promising field of research. Current studies are also exploring the potential of combining STING agonists with other therapies to improve outcomes for gastric cancer patients. However, further clinical trials and mechanistic studies are required to validate the mechanisms and potential value of STING-based treatments for gastric cancer. These efforts will provide a more solid scientific foundation for developing more effective treatment strategies for gastric cancer.

## Figures and Tables

**Table 1:** Role of cGAS-STING pathway in gastric cancer.

Sample Source (Cells, Animal Model or Clinical Patients)	Specific Information	Expression Level of cGAS-STING	Function of cGAS-STING	Reference
mice	Helicobacter pylori infection	The expression of STING protein increased	Suppression of infection	[48]
	Xenograft tumor	STING expression was low	Anlotinib activates STING and improves the efficacy of anti-PD-L1	[53]
	MFC	STING expression was low	Radiation therapy activates STING, thereby promoting the expression of PD-1/PD-L1	[54]
cells	BGC823, AGS, and SGC7901	STING expression was low	Metformin activates STING to inhibit cell proliferation and invasion	[55]
	AGS and HS746T	STING expression was low	Anlotinib activates STING and inhibits tumor cell proliferation and invasion	[53]
clinical patients	no	STING expression was increased in tumor tissue of patients	STING-positive patients had better TNM stage and OS	[48]
	EBV infection	STING expression was increased in tumor tissue of patients with EBV positive gastric cancer	STING positive expression showed better OS rate regardless of EBV infection	[49]
	Metastatic ovarian carcinoma	The expression of TBK1 and IRF3 was low	Patients with lower M2GO, which includes TBK1 and IRF3, had worse outcomes	[51]
	HER-2	The expression of STING was lower in HER2-positive patients	Patients with lower STING expression had worse prognosis	[52]

TBK1 tank-binding kinase 1; IRF3 interferon regulatory factor 3; HER2 human epidermal growth factor receptor 2; PD-1 programmed death-1; PD-L1 Programmed cell death ligand 1; EBV Epstein-Barr virus; OS overall survival; MFC mouse forestomach carcinoma.

**Table 2:** Types and characteristics of STING agonists.

Type	STING Agonist	Advantage	Insufficiency	Reference
CDN	ADU-S100/MIW815, MK-1454, SB11285, BMS-986301, BI-1387446, IMSA-101, JNJ-67544412, et al.	It belongs to natural ligand and has obvious effect; it is suitable for intratumoral drug delivery therapy	It has poor stability, poor cell targeting, and low cell uptake efficiency	[59,60]
NCDN	DMXAA, MK-2118, GSK-3745417, SNX281, TAK-676, E7766, SR-717, RVU3128603, et al.	It has better oral absorption prospects and lower production costs	The clinical trial of DMXAA failed, and the clinical trial results of other drugs were not clear	[61]

CDN cyclic dinucleotides; NCDN non-cyclic dinucleotides.

**Table 3:** Clinical trials of STING agonists combined with other antitumor drugs.

Type of Agonists	Name	Tumor	Combination Therapy	Phase	NCT Code
CDN	MIW815	Advanced/Metastatic solid tumors or lymphomas	Spartalizumab(PD-1 Inhibitor)	I	NCT03172936
		Head and neck squamous cell carcinoma	Pembrolizumab(PD-1 Inhibitor)	II	NCT03937141
	MK-1454	Advanced/Metastatic solid tumors or lymphomas	Pembrolizumab	I	NCT03010176
		Head and neck squamous cell carcinoma	Pembrolizumab	II	NCT04220866
	BMS-986301	Advanced solid tumors	Nivolumab	I	NCT03956680
	SB-11285	Advanced solid tumors	Atezolizumab (PD-L1 Inhibitor)	I	NCT04096638
NCDN	DMXAA	Non-small cell lung cancer	Docetaxel or PC	III	NCT00738387, NCT00662597, NCT00832494
		Advanced solid tumors	Docetaxel or PC	I	NCT01290380, NCT01278849, NCT01240642, NCT01031212
	MK-2118	Advanced/Metastatic solid tumors or lymphomas	Pembrolizumab	I	NCT03249792
	GSK-3745417	Advanced solid tumors	Dostarlimab	II	NCT03843359
	SNX281	Advanced/Metastatic solid tumors or lymphomas	Pembrolizumab	I	NCT04609579
	TAK-676	Advanced solid tumors	Pembrolizumab	I	NCT04420884
		Non-small cell lung cancer	Pembrolizumab	I	NCT04879849

CDN cyclic dinucleotides; NCDN non-cyclic dinucleotides; PD-1 programmed cell death protein 1; PD-L1 Programmed cell death ligand 1.

## Data Availability

Not applicable.

## Abbreviations

TME	Tumor microenvironment
cGAS	cyclic GMP–AMP synthase
STING	stimulator of interferon genes
DAMP	damage-associated molecular pattern
dsDNA	double-stranded DNA
cGAMP	cyclic GMP-AMP
ATP	Adenosine Triphosphate
CTD	C-terminal nucleotide transferase domain
ER	Endoplasmic reticulum
CTT	C-terminal tail
ERGIC	endoplasmic reticulum Golgi intermediate chamber
TBK1	tank-binding kinase 1
IKK	inhibitor of kappa B kinas
IRF3	interferon regulatory factor 3
IFN	interferon
NF-kB	nuclear factor kappa B
TAK1	TGF-beta-activated kinase 1
IKK	I kappa B kinase
NK	cells natural killer cells
CRC	colorectal cancer
CDN	cyclic dinucleotides
NCDN	non- cyclic dinucleotides
OS	overall survival
HER2	human epidermalal growth factor receptor receptor 2
PD-1	programmed death-1
PD-L1	Programmed cell death ligand 1
MFC	mouse forestomach carcinoma
